# Knowledge and attitudes of medical students regarding human papilloma virus infection and vaccine: cross-sectional study from Jordan

**DOI:** 10.3389/fcimb.2025.1657090

**Published:** 2025-09-18

**Authors:** Hana Taha, Taher Alhawamdeh, Sireen M. Alkhaldi, Rania Ali Albsoul, Abdallah Al-Ani, Suhib Awamleh, Amin Y. Al-Maayeh, Arwa Qaqish, Ameen Mahmoud, Diana Abu-Surrah, Vanja Berggren

**Affiliations:** ^1^ Department of Family and Community Medicine, School of Medicine, The University of Jordan, Amman, Jordan; ^2^ Department of Neurobiology, Care Science and Society, Karolinska Institutet, Stockholm, Sweden; ^3^ Primary Healthcare Department, King Khalid Hospital, Kharj, Saudi Arabia; ^4^ Office of Scientific Affairs and Research, King Hussein Cancer Center, Amman, Jordan; ^5^ Faculty of Medicine, The Hashemite University, Zarqa, Jordan; ^6^ Department of Biology and Biotechnology, Faculty of Science, The Hashemite University, Zarqa, Jordan

**Keywords:** human papilloma virus, Jordan, medical education, knowledge, vaccine

## Abstract

**Background:**

As of the present moment, Jordan is yet to incorporate cervical cancer screening in its cancer control program nor advocates for vaccines. This paper aims to examines the perceptions and attitudes of medical students towards HPV and its vaccine.

**Methods:**

We conducted a cross-examination of HPV knowledge and vaccine uptake among medical students across the period between January and March 2024. Participants completed a questionnaire developed and validated by the existing literature. The questionnaire was composed of 4 domains pertaining to sociodemographic, knowledge of HPV, knowledge of HPV vaccine, and attitudes. Medical students were conveniently sampled from Jordan’s six public medical schools. Predictors to self-vaccinate, recommending vaccination to friends/family, and recommending vaccination to patients were examined using a binary logistic regression model. All analyses were conducted on R version (4.3.3).

**Results:**

A total of 473 medical students were included in the final analysis. On a scale of 12 and 8, mean HPV and vaccine knowledge scores were 5.4 ± 3.1 and 2.9 ± 1.9, respectively. Knowledge of HPV and its vaccine were significantly higher among females, students in their clinical years, and those with self-perceived understanding of HPV (all p <0.05). Intention to self-vaccinate against HPV was predicted by higher HPV and vaccine knowledge scores. Male participants were significantly less likely to self-vaccinate compared to females (OR: 0.61; 95%CI: 0.40 – 0.91). Similarly, higher HPV and vaccine knowledge scores were associated a higher likelihood to recommend the vaccine to family or patients. On the other hand, male participants were significantly less likely to recommend the vaccine to patients compared to their female counterparts (OR: 0.62; 95%CI: 0.40 – 0.95).

**Conclusion:**

The study implies that the overall awareness and attitudes regarding HPV and its vaccine is alarmingly poor among medical students. Moreover, there exists a gender difference in the knowledge and attitudes favoring females. Concerned policy makers and institutions should strive to improve vaccine awareness and uptake through informational, behavioral, and environmental interventions. Moreover, medical students should be well equipped to tackle HPV vaccine hesitancy through curricular reforms, targeted training, and increased exposure to public vaccine promotional efforts.

## Background

Human papillomaviruses (HPV) are double-stranded DNA non-enveloped viruses that are associated with carcinogenesis (Abdelaziz et al., 2025). HPV infects squamous epithelial cells in the skin and mucosa and usually causes benign warts. However, persistent infection with high-risk oncogenic HPV strains causes cervical, anal, vulvar, vaginal, penile and oropharyngeal cancers.

(Abdelaziz et al., 2025). HPV have almost 200 genotypes and are categorized as high- or low-risk per their capacity to cause cancer. HPV is commonly transmitted through sexual or skin-to-skin contact among other mediums (Albayat et al., 2024). While most HPV infections resolve spontaneously, others may persist causing skin lesions, genital warts, or cancer (de Sanjosé et al., 2018). With more than 300 million affected women worldwide, HPV infection is considered a global public health concern (Kombe Kombe et al., 2021). In fact, 12% of women with normal cervical cytology are carrier of HPV.

According to the World Health Organization (WHO) global statistics, HPV is responsible for an estimated 620,000 and 70,000 cancer cases in women and men, respectively (World Health Organization, 2024). Of these cancers, cervical cancer is associated with the most significant morbidity and mortality as it is the fourth cause of death across all female cancers (Sung et al., 2021). Fortunately, the risk of HPV infections can be mitigated by vaccines. As of writing this study, three vaccine types have been approved to prevent HPV genotypes implicated in cervical cancer (Wang et al., 2020). Current evidence points to the high safety and efficacy of these commercially available HPV vaccines despite the need for long-term studies (Porras et al., 2020; Alsanafi et al., 2023).

In the Middle East and North Africa (MENA) region, there are significant barriers to the uptake of the HPV vaccine. These include financial restraints, poor infrastructure, competition with other vaccines, and general lack of awareness (Abdelaziz et al., 2025). However, these barriers are context sensitive to the cultural and religious norms characterizing the MENA nations. Such factors could reduce the effectiveness of vaccination campaigns or increase resistant to its uptake among both the general public and medical students. Nonetheless, the literature shows that gaps in HPV knowledge exists also for healthcare professionals (Sallam et al., 2022).

While exact numbers on HPV infections and HPV-associated cancers in Jordan are currently unavailable, one study of 650 cases referred from gynecology clinics found HPV in 25.7% of native Jordanians’ samples (Annab et al., 2025). Estimates suggest that 115 women are diagnosed with cervical cancer, and 71 women die from the disease every year in Jordan (HPV Information Centre). Considering that 90% of cervical cancer cases are preventable through HPV vaccination (CDC, 2025), this emphasizes the vital role of vaccination as a preventive measure.

Currently, Jordan lacks a national screening program for cervical cancer nor includes HPV as part of its national immunization policy. Moreover, this is complicated by the lack of reliable data and updates on the prevalence of HPV across the kingdom (Mahafzah et al., 2008; Qaqish et al., 2023). Therefore, this paper aims to examine the understanding and awareness and the attitudes towards HPV infection among Jordanian medical students and the factors influencing vaccine uptake.

## Methods

### Study settings

Medical education in Jordan has evolved in the last 50 years with the establishment of several medical schools and teaching hospitals around the country. Currently in Jordan, there are approximately 10,000 medical students, with 1500 graduates per year (Sarhan et al., 2025). This study recruited students from all six public medical schools in Jordan (University of Jordan, Hashemite University, Balqa Applied University, Jordan University of Science and Technology, Mut’ah University, and Yarmouk University). Participants were from both basic and clinical years. The choice of medical students was purposive, given their role as future practitioners, highlighting their importance in raising awareness, promoting vaccine advocacy, and public health interventions related to HPV in Jordan.

### Study design

A cross-sectional descriptive study was conducted between January and March 2024. This design was selected to provide a snapshot of the current awareness and attitudes towards HPV because it is practical and cost-effectiveness for establishing baseline data on HPV knowledge and vaccine uptake.

### Measurement tool

The questionnaire for this study (**Supplementary 1**) was developed based on existing literature (Khalil et al., 2023; Nesser and Ayodele, 2023; Qaqish et al., 2023; Albayat et al., 2024). The adopted questions were forward translated from English to Arabic by bi-lingual members of the research team. An expert back translated the questionnaire from Arabic to English to ensure equivalence. The final Arabic questionnaire was evaluated by an expert committee of medical and public health experts.

The domains and items comprising the HPV and HPV vaccine knowledge scores were examined for content validity and reliability. Results from the expert committee assessment revealed a Scale – Content Validity Index for the HPV and HPV vaccine knowledge scales of 0.831 and 0.764, respectively. After ensuring content validity, the questionnaire was pilot tested on 20 medical students for confirm clarity, understandability, relevance, and reliability prior to initiating data collection. Cronbach’s α values for the HPV knowledge and HPV vaccine knowledge domains were 0.756 and 0.692, respectively.

The final tool was comprised of 4 domains pertaining to sociodemographic characteristics, knowledge regarding HPV infection and vaccine, attitudes towards HPV infection and vaccine. The sociodemographic domain included among others questions about sex, age, country of origin, marital status, as well as the level of study and the university attended by each participant. The 2^nd^ domain assessing the participants’ knowledge of HPV infection consisted of 14 items, each with a single correct answer, and participants answers were scored based on the number of correct responses they provided. The 3^rd^ domain, which evaluated participants’ knowledge of the HPV vaccine, included eight items and followed the same scoring protocol as the previous section. The final domain included questions assessing the student’s willingness to self-vaccinate, recommending the vaccine to family members, or recommending it to patients, as well as questions about the factors that could influence these decisions.

### Sample size and data collection

Given the descriptive nature of our study and the logistical challenges of surveying medical students with demanding schedules, convenience sampling provided a practical and cost-effective solution. However, we acknowledge that convenience sampling may introduce some limitations, and we discussed these in a subsection of the discussion.

A minimum of 384 responses was determined using Cochran’s formula (N = (Z^2^ p(1-p)/e^2^). We set the confidence level at 95% (Z = 1.96), specified a maximum margin of error of ±5% (E = 0.05), and used the conservative proportion p = 0.50 to ensure the largest required sample size. A total of 473 responses were included in the study to ensure adequate representation of the study population.

The data was collected using an Arabic self-administered online survey, which was placed on Google Forms and disseminated through the official WhatsApp, Facebook, and Microsoft Teams groups for all six medical schools included in the study. The inclusion criteria were: Full-time registered undergraduate medical students aged 18 years and above from all six public medical schools in Jordan. We targeted medical students from both the basic years (1^st^ – 3^rd^ year) and the clinical years (4^th^ – 6^th^ year) to ensure a comprehensive representation of different stages in medical education. The exclusion criteria were medical students aged less than 18 years and unregistered medical students.

### Ethical considerations

This study was approved by Institutional Review Boards (IRB) of the Hashemite University (No. 22/7/2021/2022) and the University of Jordan (No.446/2024/2025). All the participants provided a written consent prior to participating in this study. The researchers adhered to strict ethical guidelines and ensured the anonymity of all participants.

### Statistical analysis

Data was imported in and analyzed using R (version 4.3.3). Continuous variables were summarized as mean and standard deviation, while categorical variables were summarized as counts and percentages. Associations between categorical variables were assessed using the chi-squared test. Mean differences (MD) in continuous variables were assessed using the independent sample-t test. Predictors to self-vaccinate, recommending vaccination to friends/family, and recommending vaccination to patients were examined using a binary logistic regression model. Each predictor variable was expressed using odds ratios (OR) and their associated 95% confidence interval (95%CI). Figures were produced using GraphPrism 8.3.4. A p-value of < 0.05 was considered statistically significant.

## Results

### Characteristics of the included sample

A total of 473 medical students were included in the final analysis with a male-to-female ratio of 0.76-to-1.00 (43.3%-to-56.7%). The majority of participating medical students were single (97.5%), of Jordanian origin (91.1%), and studying at the basic medical sciences level (64.5%). While 53.5 were older than 20 years, 46.5% were younger than 20 years of age. In terms of sampled medical schools, most participants were recruited from the Hashemite University (68.7%), University of Jordan (13.1%), and Yarmouk University (9.7%). The other participants were recruited from Al-Balqa’ Applied University (5.1%), Jordan University of Science and Technology (3.0%), and Mutah University (0.4%). When asked about their sources on HPV-related knowledge, medical education (56.4%), self-reading (23.3%), and social media (12.9%) were the most common. **Table 1** demonstrates the characteristics of study participants.

**Table 1:** Characteristics of included participants.

Variable	Category	N	%
Sex	Male	205	43.3
	Female	268	56.7
Year of study	1^st^	122	25.8
	2^nd^	125	26.4
	3^rd^	58	12.3
	4^th^	62	13.1
	5^th^	65	13.7
	6^th^	41	8.7
Country of origin	Jordanian	431	91.1
	Non-Jordanian	42	8.9
Marital status	Married	12	2.5
	Single	461	97.5
University	Balqa’ Applied University	24	5.1
	Hashemite University	325	68.7
	University of Jordan	62	13.1
	Jordan University of Science and Technology	14	3.0
	Mutah University	2	0.4
	Yarmouk University	46	9.7

### Knowledge of HPV

Among the included medical students, 72.3% reported self-perceived familiarity with HPV, while 22.8% did not, and 4.9% being neutral. Of all included participants, only 27.5% believed that HPV is common in Jordan, 35.7% being neutral, and 36.8% disagreeing. In terms of infection susceptibility, 68.7% of students believe that HPV could affect both males and females, while 15.6% and 2.3% stated that it can only affect females or males, respectively. Moreover, 58.6% of participants recognized that HPV could be asymptomatic and 54.8% knew that sexual intercourse is not the only medium for virus transmission. Among the latter, skin-to-skin transmission was recognized by 45.0%, skin-to-mucosa transmission by 56.1%, mother-to-fetus transmission by 48.6%, transmission through contaminated medical equipment by 49.1%, transmission through contaminated water by 20.1%, and transmission through self-inoculation by 36.6%.

When asked about the most important risk of HPV in females, cancer (52.6%) was most perceived compared to genital warts (18.8%) or skin warts (6.6%). On the other hand, when asked about the most important risk of HPV in males, 38.3% noted genital warts, 14.4% reported skin warts, and only 13.5% considered cancer. In terms of HPV treatment, 55.8% of participants believed that it can be cured compared to 30.0% neutral participants and 14.2% denying such possibility. Finally, only 27.9% of participants correctly recognized that cervical cytology should be conducted once every three years. **Table 2** demonstrates the responses to HPV knowledge items.

**Table 2:** Responses to items pertaining to knowledge of HPV.

Question	Correct response	N	%
HPV is common in Jordan	No	174	36.8
Who are susceptible to HPV infections?	Both males and females	325	68.7
HPV infection can be asymptomatic	Yes	277	58.6
HPV is transmitted through sexual intercourse only	No	259	54.8
Skin-to-skin contact is a route for HPV transmission	Yes	173	44.8
Skin-to-mucosa contact is a route for HPV transmission	Yes	217	56.4
Mother-to-fetus contact is a route for HPV transmission	Yes	197	50.5
Contaminated medical equipment is a route for HPV transmission	Yes	193	50.8
Contaminated water is a route for HPV transmission	No	121	32.8
Self-inoculation is a route for HPV transmission	Yes	139	37.2
The major risk of HPV in females is	Cancer	249	52.6
The major risk of HPV in males is	Cancer	64	13.5
Can HPV be treated	No	67	14.2
How often should cervical cytology be performed	Once every 3 years	132	27.9

The mean knowledge score for HPV was 5.37 ± 3.15 ranging from 0 to 12. Participants in their clinical years had significantly higher knowledge scores compared to their counterparts in basic years (MD: -2.92; 95%CI: -3.45 – -2.38). Similarly, female participants had significantly higher knowledge scores than their male counterparts (MD: 1.07; 95%CI: 0.51 – 1.64). Finally, participants with perceived familiarity of HPV had higher knowledge scores compared to their counterparts (MD: -3.84; 95%CI: -4.37 – -3.31). There were no significant differences in knowledge scores in terms of nationality or marital status (p = 0.142 and 0.753, respectively).

### Knowledge of HPV vaccine

Among included participants, 49.0% noted a self-perceived familiarity with HPV vaccine, 30.9% did not, while 20.1% were neutral. The majority of participants did not know if the vaccine is protective for persons infected with HPV, while 35.9% responded that it isn’t. About 27.7% believed that individuals with HPV-related genital warts are indicated to take the vaccine. Similarly, only 25.2% believe that there exist indications for vaccinating young males. While 41.9% recognized the most optimal time for HPV vaccination, 33.6% were neutral, 15.6% reported “after marriage/sexual activity”, and 8.9% recommended vaccination of children under the age of 8 years. In terms of vaccine dosage, an optimal dose of three was recognized as the most optimal by 55.4% of participants. The majority of participants believed that persons between the ages of 14 to 45 should be vaccinated against HPV-related disease (56.9%). Nonetheless, only 16.9% were able to recognize the estimated percentage of prevented cervical cancers by the vaccine at 90%. Similarly, only 31.3% of participants knew that the HPV vaccine is available in Jordan. **Table 3** demonstrates the responses to HPV vaccine knowledge items.

**Table 3:** Responses to items pertaining to knowledge of HPV vaccine.

Question	Response	N	%
The vaccine is protective for persons already infected with HPV	No	170	35.9
Persons suffering from HPV-related genital warts are indicated to be vaccinated against HPV	Yes	131	27.7
There are indications for vaccinating young males against HPV	Yes	119	25.2
What is the best time to get vaccinated against HPV	Children aged 9 – 13 or before marriage/sexual activity	198	41.9
How many doses of HPV vaccine are recommended in a life time	Three doses	262	55.4
Can individuals aged 14 to 45 be vaccinated to protect against genital warts or cancer	Yes	269	56.9
The vaccine is available in Jordan	Yes	148	31.3
What is the estimated percentage of cervical cancers prevented by the HPV vaccine?	90%	80	16.9

The mean knowledge score of HPV vaccine was 2.91 ± 1.88 ranging from 0 to 8. Participants in their clinical years had significantly higher vaccine knowledge scores compared to their counterparts in basic years (MD: -1.45; 95%CI: -1.79 – -1.12). Similarly, female participants had significantly higher vaccine knowledge scores than their male counterparts (MD: 0.34; 95%CI: 0.01 – 0.68). Finally, participants with perceived familiarity of HPV had higher vaccine knowledge scores compared to their counterparts (MD: -1.62; 95%CI: -1.92 – -1.31). There were no significant differences in vaccine knowledge scores in terms of nationality or marital status (p = 0.151 and 0.764, respectively).

### Attitudes towards HPV vaccination

**Figure 1:**
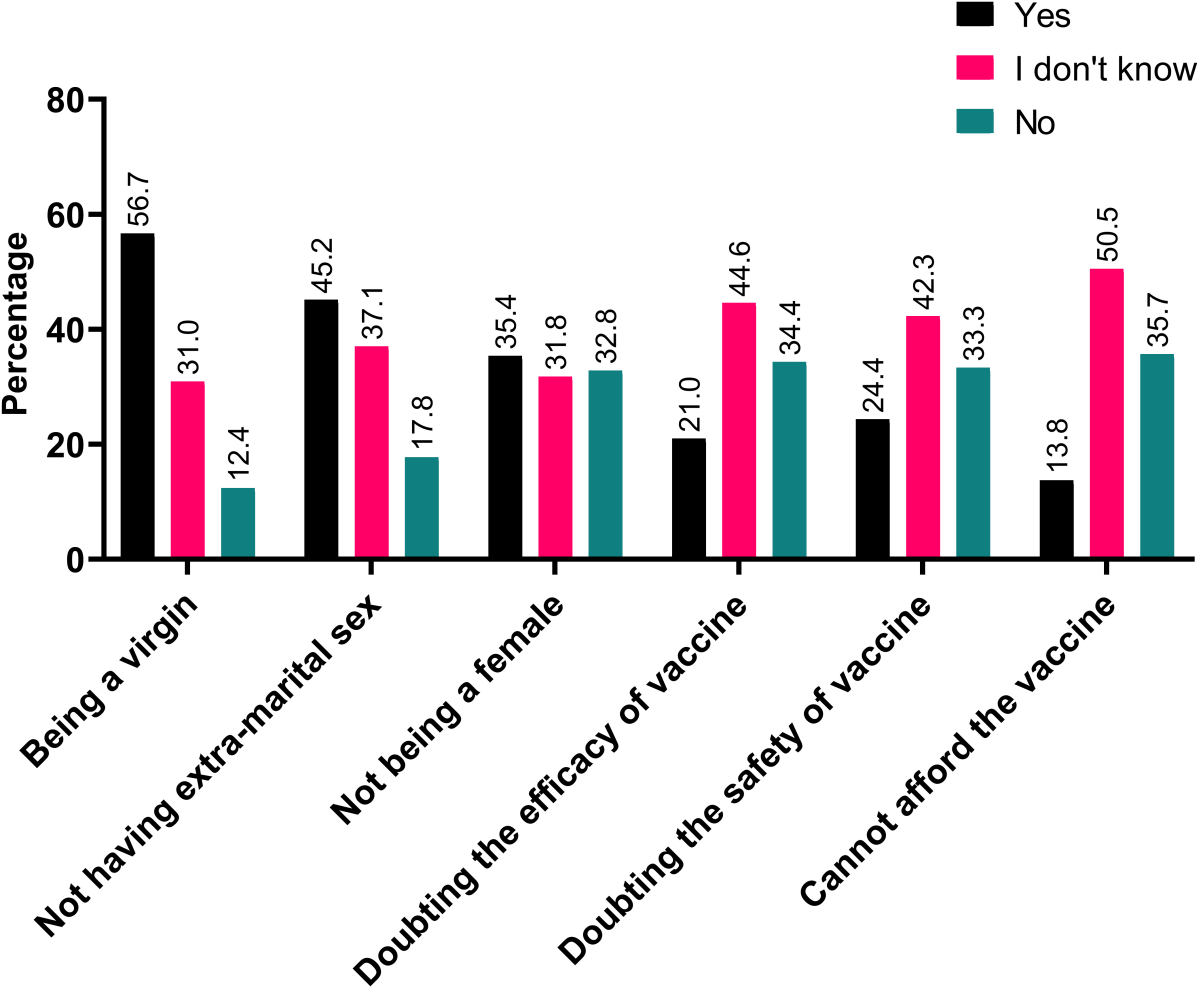
Most commonly reported barriers for self-vaccination against HPV.

About 42.1% of participants believe that it should be obligatory to vaccinate against HPV while 24.7% disagreed with such notion. When asked about the possibility of self-vaccination, 39.3% would take the HPV vaccine if its available for free in Jordan. Among those that declined such possibility, being a virgin (56.7%), having no extra marital sex (45.2%), and not being a female (35.4%) were the most common reasons for rejecting the vaccine (**Figure 1**).

**Figure 2:**
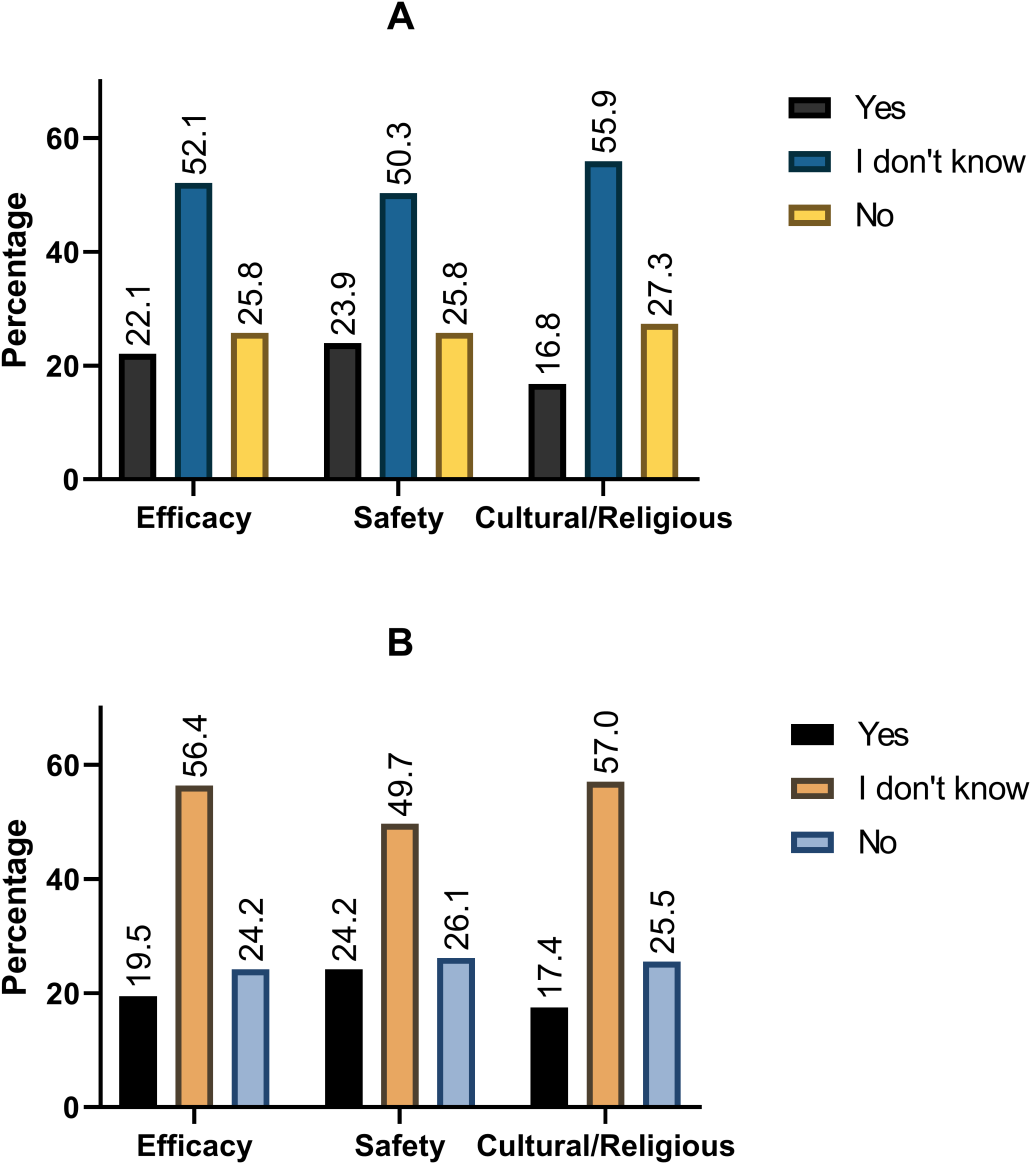
Most commonly reported barriers to recommending HPV vaccines to **(A)** friends and family and **(B)** patients.

Moreover, 56.7% would recommend the vaccine to a family member, while 59.4% would recommend it to patients. Among those that would not recommend the vaccine to a family member, doubts about the vaccine safety (23.9%) and efficacy (22.1%) were prevalent (**Figure 2**). Similarly, doubts over vaccine safety were the most common barrier to recommending the vaccine to patients (24.2%).

### Predictors of intention to vaccinate

Intention to self-vaccinate against HPV was predicted by higher HPV knowledge scores (OR: 1.12; 95%CI: 1.03 – 1.22) and higher vaccine knowledge scores (OR: 1.20; 95%CI: 1.06 – 1.36). Male participants were significantly less likely to self-vaccinate compared to females (OR: 0.61; 95%CI: 0.40 – 0.91). Similarly, participants with higher HPV knowledge scores (OR: 1.14; 95%CI: 1.05 – 1.23), higher HPV vaccine knowledge scores (OR: 1.26; 95%CI: 1.10 – 1.43), and are in their clinical studies (OR: 1.68; 95%CI: 1.05 – 2.70) were more likely to recommend the HPV vaccine to their family. Conversely, single participants were less likely to recommend that vaccine to family (OR: 0.18; 95%CI: 0.03 – 0.93). Finally, participants with higher HPV knowledge scores (OR: 1.16; 95%CI: 1.07 – 1.26), higher HPV vaccine knowledge scores (OR: 1.29; 95%CI: 1.12 – 1.47), and are in their clinical studies (OR: 1.80; 95%CI: 1.10 – 2.95) were more likely to recommend the HPV vaccine to their patients. On the other hand, male participants were significantly less likely to recommend the vaccine to patients compared to their female counterparts (OR: 0.62; 95%CI: 0.40 – 0.95). **Table 4** demonstrates the binary logistic regression model for the predictors of all three intentions with regards to the HPV vaccine.

**Table 4:** Binary logistic regression of the predictors of intention to self-vaccinate, recommending HPV vaccine to family, and recommending HPV vaccine to patients.

	Self-vaccinate		Vaccinate family		Vaccinate patients	
Variable	OR	95%CI	OR	95%CI	OR	95%CI
HPV knowledge score	**1.12**	**1.03 – 1.22**	**1.14**	**1.05 – 1.23**	**1.16**	**1.07 – 1.26**
Vaccine knowledge score	**1.20**	**1.06 – 1.36**	**1.26**	**1.10 – 1.43**	**1.29**	**1.12 – 1.47**
Stage of study (i.e., Clinical)	1.49	0.96 – 2.34	**1.68**	**1.05 – 2.70**	**1.80**	**1.10 – 2.95**
Sex (i.e., Male)	**0.61**	**0.40 – 0.91**	0.70	0.46 – 1.06	**0.62**	**0.40 – 0.95**
Marital Status (i.e., Married)	0.18	0.21 – 1.47	**0.18**	**0.03 – 0.93**	0.31	0.07 – 1.33
Country of Origin (i.e., Jordanian)	0.69	0.34 – 1.39	1.09	0.53 – 2.27	1.36	0.65 – 2.84

Bold OR values lie within 95% CI.

## Discussion

In this study, we demonstrated an alarming gap in HPV and vaccine knowledge among medical students. Only 72.3% were aware of the disease, while only 49.0% were aware of the vaccine. Significant knowledge gaps exist for epidemiology, treatment, transmission, and vaccination for HPV. Intention to self-vaccinate was lower than willingness to recommend the vaccine to family or patients. Factors associated with intention to vaccinate or recommending the vaccine were female gender, higher knowledge scores, and being in the clinical phase of undergraduate medical studies.

These findings, while alarming, are parallel to the existing body of literature. Poor HPV awareness, vaccine knowledge, and subpar intention to vaccinate were documented among university students in Saudi Arabia, Kuwait, Qatar, Iran, Morocco, Oman, Bahrain, and India (Ghojazadeh et al., 2012; Rashid et al., 2016; Alshammari and Khan, 2022; Bencherit et al., 2022; El Mansouri et al., 2022; Yacouti et al., 2022; Alsanafi et al., 2023; Alghalyini et al., 2024; Cheema et al., 2024). Interestingly, a similar trend was found across studies focusing on exclusively medical students (Mehta et al., 2013; Farsi et al., 2021; Haddad et al., 2022; Abdelaziz et al., 2025). A reliable explanation for this phenomenon originates in the sociocultural characteristics of the Middle East and North Africa (MENA) region. The MENA region is a conservative culture dominated by Islamic practices (Abi Jaoude et al., 2018). While the earlier could lead individuals to believe that safer sexual practices may lead to promiscuity and tarnish family prestige, the latter considers any extra-marital sexual activities to divinely prohibited; therefore, there exists a regional taboo on discussing any matters related to sexual activity (Hamdi, 2017).

On a more locally focused note, the Hashemite Kingdom of Jordan is known for an extremely limited cancer screening and prevention program, which only pertains to breast cancer (Al-Shamsi et al., 2022). Screening for cervical cancers through “pap smears”, prevention through vaccines, or even raising awareness through campaigns are yet to be complied into a policy despite somewhat shy efforts to promote prevention by private institutions and individuals. In fact, in a country where the minimum wage is about 410$, the HPV vaccine is estimated to cost 250$. Therefore, vaccination is only limited for those with high-end resources. This is particular importance as vaccine costs represent the most commonly reported factor for HPV vaccine resistance (Gamaoun, 2018). In response to cost barriers, Murewanhema et al., recommends enhanced vaccine cost coverage by insurance providers, the development of local infrastructure for the production of HPV vaccine, and embedding vaccine administration programs within schools, all of which serves of lower costs within resource limited countries (Murewanhema et al., 2024). While Jordan does not qualify for the Global Alliance for Vaccines and Immunizations (GAVI) monetary support, concerned policy makers could attempt negotiating cost-effective pricing of vaccines for the economically disadvantaged sectors of the population.

Across the literature, females demonstrated significantly higher knowledge of HPV and its associated vaccine compared to males (Rashid et al., 2016; Dai et al., 2022; Teeluck et al., 2022; Alghalyini et al., 2024; Chen et al., 2024). This is attributed to the stereotypical portrayal of HPV and cervical cancer. It could be also explained by the Health Belief Model, as the stereotypical portrayal could enhance the perceived susceptibility of disease among females compared to males (Al-Sabbagh et al., 2021; Oh et al., 2023). However, this is not entirely the case for some populations as Rashid et al., demonstrates that despite higher levels of awareness and knowledge of HPV, females were less likely to vaccinate compared to their male counterparts (Rashid et al., 2016). It is believed that such hesitancy stems from societal, religious, and prejudice ideas as well as poor socioeconomic status.

There is a consistent trend throughout the literature for a higher intention to vaccinate among those with biological/medical backgrounds, university degrees, or have received dedicated sexual education courses (Rashid et al., 2016; Dai et al., 2022; El Mansouri et al., 2022; Yacouti et al., 2022; Chen et al., 2024). The Theory of Planned Behavior (TPB) encapsulates such findings as it demonstrates that one’s attitudes and subjective norms affect behavior indirectly through behavioral intentions which are considered a direct precursor of behavior (Ajzen, 1991); this phenomenon is well documented for HPV vaccination intentions (Shah et al., 2021; Orji et al., 2025). Therefore, should all environmental factors be constant, better education will encourage individuals to pay more attention to potential diseases and have positive attitudes toward taking meaningful measures to either prevent the problem or cope with it. Unfortunately, while a plethora of studies indicate gaps within their sexual education programs at both the secondary and graduate levels, such courses are not provided within Jordanian institutions nor even attempted. This is owed to the complex interplay of Jordanian socio-religious culture that could stigmatize any efforts involving raising awareness on sex-related matters (Othman et al., 2022).

Moreover, a number of studies throughout the MENA region documented the role of conspiracy theories in augmenting vaccine hesitancy/resistance. Embracing vaccine conspiracies and vaccine resistance was documented for the COVID-19 vaccine, influenza vaccine, and the HPV vaccine (Knobel et al., 2022; Pertwee et al., 2022; Alsanafi et al., 2023; Alshehri and Sallam, 2023). This may have an unforeseen impact on participants’ intentions as nearly 44% of the current sample obtain their HPV-related knowledge through social circles, social media, or self-reading. The latter is particularly important as reliance on internet-based sources was linked with lower intentions to self-vaccinate against HPV (Alsanafi et al., 2023).

Jordan and so does many of the MENA countries require extensive efforts in promoting HPV knowledge and vaccine uptake. According to the Community Guide’s Data Abstraction (CGDA) framework, improving the status of HPV awareness and vaccine uptake must rely on through phases of evidence-based interventions: informational, behavioral, and environmental (Cheema et al., 2024). At the informational level, concerned authorities must dedicate resources to the development and dissemination of media campaigns to boost knowledge about HPV and its vaccine. A successful example includes the “Healthy China 2023” plan which is attributed as a significant factor in improving the public’s collective knowledge on HPV vaccination and cervical cancer screening (Wang et al., 2021; Chen et al., 2024). These campaigns should be mediated through healthcare workers, religious leaders, and social media platforms. The latter is especially relevant as it allows for an active role of susceptible individuals when interacting with information, ultimately contributing to informed health decision-making (Wang et al., 2021). Such plans should target not only susceptible individuals but also their immediate family as a plethora of studies demonstrated the role of parental encouragement and education in increase HPV vaccine uptake (Abdullahi et al., 2020; Rani et al., 2022).

At the behavioral level, the patient-healthcare provider relationship must be revamped to further increase vaccine uptake and general knowledge through providing cues to action (Al-Sabbagh et al., 2021; Al-Ani et al., 2024). Examples of these interventions include peer group advocacy, provider-led reminders to get vaccinated, and home visits. Reno et al., emphasizes that healthcare workers should also be trained and equipped with the necessary communication skills as to provide a clear message about the risks of HPV and building rapport with susceptible individuals (Reno et al., 2019). Improving the trust between patients and providers will likely reduce the impact of misinformation and contribute to the development of a more informed patient. Finally, environmental policies should be adopted at the national level. Governments are encouraged to include HPV vaccine as part of their national vaccination policy or subsidize their costs, making them more accessible. These policies, while cost intensive at an initial glance, may provide significant economic savings in the long-term by preventing HPV-related disease costs.

At the level of medical students, policy makers and university administrations should revise and re-develop their existing curriculums. Medical students, which are considered future practitioners, should be well equipped to prevent HPV across both themselves and their future patients. The content, approach, and style of medical educational materials could be improved as to provide these students with the necessary evidence and theoretical basis for circumventing the disease (Solis-Torres et al., 2024; Abdelaziz et al., 2025). Moreover, any changes made should take into consideration the stigma around sexually transmitted diseases which may influence attitudes of even medical students towards the promotion of the vaccine (Abdelaziz et al., 2025). Medical students should be encouraged and trained to approach these sensitive topics educating the public and addressing hesitancy.

In light of what’s above, our findings should be interpreted within the context of a number of limitations. These include the cross-sectional nature of study’s design, close-ended questionnaire, and due to logistics, inability of the questionnaire to account for all factors affecting HPV knowledge. Participants may also demonstrate social desirability bias or a neutral bias which might have distorted responses or compromised data accuracy. Additionally, due to the fact that this study is comprised of medical students only, its results cannot be generalized to other university students in non-medical fields or even other medical fields such as nursing or pharmacy. The use of convenience sampling and the over-representation of students from the Hashemite University may also limit the external validity of our findings to all medical students. Certain demographics of students may have been missed by the sampling strategy and since each Jordanian University represents a number of Jordanian prefectures, the over-representation of students from one university may produce results that may not be replicable in other Jordanian institutions. Finally, due to the different answer scales for some questions, the questionnaire was not subjected to an exploratory factor analysis to quantitatively measure its construct validity.

## Conclusion

The study implies that the overall awareness and attitudes regarding HPV and its vaccine is alarmingly poor among medical students. Moreover, there exists a gender difference in the knowledge and attitudes favoring females. Our findings call for an urgent need for qualitative research to explore the barriers and facilitators to vaccination. This is necessary for designing multi-faceted, context sensitive, evidence-based interventions at the informational, behavioral, and environmental levels from all concerned authorities to improve knowledge of HPV among young adults and increase vaccine uptake.

## Data Availability

The raw data supporting the conclusions of this article will be made available by the authors, without undue reservation.

## References

[B1] Abdelaziz M. N. Hefnawy A. Azzam H. Reisha O. Hamdy O. (2025). Knowledge and attitude among Egyptian medical students regarding the role of human papillomavirus vaccine in prevention of oropharyngeal cancer: a questionnaire-based observational study. Sci. Rep. 15, 3767. doi: 10.1038/s41598-025-86853-8, PMID: 39885233 PMC11782598

[B2] Abdullahi L. H. Kagina B. M. Ndze V. N. Hussey G. D. Wiysonge C. S. (2020). Improving vaccination uptake among adolescents. Cochrane Database Syst. Rev. 1, CD011895. doi: 10.1002/14651858.CD011895.pub2, PMID: 31978259 PMC6984618

[B3] Abi Jaoude J. Khair D. Dagher H. Saad H. Cherfan P. Kaafarani M. A.. (2018). Factors associated with Human Papilloma Virus (HPV) vaccine recommendation by physicians in Lebanon, a cross-sectional study. Vaccine 36, 7562–7567. doi: 10.1016/j.vaccine.2018.10.065, PMID: 30420044

[B4] Ajzen I. (1991). The theory of planned behavior. Organ Behav. Hum. Decis. Proc. 50, 179–211. doi: 10.1016/0749-5978(91)90020-T

[B5] Al-Ani A. Hammouri M. Sultan H. Al-Huneidy L. Mansour A. Al-Hussaini M. (2024). Factors affecting cervical screening using the health belief model during the last decade: A systematic review and meta-analysis. Psychooncology 33, e6275. doi: 10.1002/pon.6275, PMID: 38282232

[B6] Albayat S. S. Mundodan J. M. Elmardi K. Hasnain S. Khogali H. Baaboura R.. (2024). Knowledge, attitude, and practices regarding human papilloma virus vaccination among physicians in Qatar. Womens. Health Lond. Engl. 20, 17455057241227360. doi: 10.1177/17455057241227360, PMID: 38282514 PMC10826392

[B7] Alghalyini B. Zaidi A. R. Z. Meo S. A. Faroog Z. Rashid M. Alyousef S. S.. (2024). Awareness and knowledge of human papillomavirus, vaccine acceptability and cervical cancer among college students in Saudi Arabia. Hum. Vaccines Immunother. 20, 2403844. doi: 10.1080/21645515.2024.2403844, PMID: 39377296 PMC11468045

[B8] Al-Sabbagh M. Q. Al-Ani A. Mafrachi B. Siyam A. Isleem U. Massad F. I.. (2021). Predictors of adherence with home quarantine during COVID-19 crisis: the case of health belief model. Psychol. Health Med. 0, 1–13., PMID: 33427487 10.1080/13548506.2021.1871770

[B9] Alsanafi M. Salim N. A. Sallam M. (2023). Willingness to get HPV vaccination among female university students in Kuwait and its relation to vaccine conspiracy beliefs. Hum. Vaccines Immunother. 19, 2194772. doi: 10.1080/21645515.2023.2194772, PMID: 37005342 PMC10088927

[B10] Alshammari F. Khan K. U. (2022). Knowledge, attitudes and perceptions regarding human papillomavirus among university students in Hail, Saudi Arabia. PeerJ 10, e13140. doi: 10.7717/peerj.13140, PMID: 35345591 PMC8957278

[B11] Al-Shamsi H. O. Abu-Gheida I. H. Iqbal F. Al-Awadhi A. (Eds.) (2022). Cancer in the arab world (Singapore: Springer Singapore). doi: 10.1007/978-981-16-7945-2

[B12] Alshehri S. Sallam M. (2023). Vaccine conspiracy association with higher COVID-19 vaccination side effects and negative attitude towards booster COVID-19, influenza and monkeypox vaccines: A pilot study in Saudi Universities. Hum. Vaccines Immunother. 19, 2275962. doi: 10.1080/21645515.2023.2275962, PMID: 37941437 PMC10653693

[B13] Annab A. W. Lataifeh I. M. Dajani Y. F. (2025). Human Papillomavirus in Jordan-A selective study of 650 cases. IJID. Reg. 15, 100620. doi: 10.1016/j.ijregi.2025.100620, PMID: 40213027 PMC11982494

[B14] Bencherit D. Kidar R. Otmani S. Sallam M. Samara K. Barqawi H. J.. (2022). Knowledge and awareness of Algerian students about cervical cancer, HPV and HPV vaccines: A cross-sectional study. Vaccines 10, 1420. doi: 10.3390/vaccines10091420, PMID: 36146498 PMC9505646

[B15] CDC (2025). “Cancers caused by HPV,” in Human Papillomavirus (HPV). Available online at: https://www.cdc.gov/hpv/about/cancers-caused-by-hpv.html.

[B16] Cheema S. Abraham A. Maisonneuve P. Jithesh A. Chaabna K. Al Janahi R.. (2024). HPV infection and vaccination: a cross-sectional study of knowledge, perception, and attitude to vaccine uptake among university students in Qatar. BMC Public Health 24, 2316. doi: 10.1186/s12889-024-19792-0, PMID: 39187821 PMC11348518

[B17] Chen X. Xu T. Wu J. Sun C. Han X. Wang D.. (2024). Exploring factors influencing awareness and knowledge of human papillomavirus in Chinese college students: A cross-sectional study. Hum. Vaccines Immunother. 20, 2388347. doi: 10.1080/21645515.2024.2388347, PMID: 39140222 PMC11326451

[B18] Dai Z. Si M. Su X. Wang W. Zhang X. Gu X.. (2022). Willingness to human papillomavirus (HPV) vaccination and influencing factors among male and female university students in China. J. Med. Virol. 94, 2776–2786. doi: 10.1002/jmv.27478, PMID: 34825712 PMC9299831

[B19] de Sanjosé S. Brotons M. Pavón M. A. (2018). The natural history of human papillomavirus infection. Best Pract. Res. Clin. Obstet. Gynaecol. 47, 2–13. 28964706 10.1016/j.bpobgyn.2017.08.015

[B20] El Mansouri N. Ferrera L. Kharbach A. Achbani A. Kassidi F. Rogua H.. (2022). Awareness and knowledge associated to Human papillomavirus infection among university students in Morocco: A cross-sectional study. PloS One 17, e0271222. doi: 10.1371/journal.pone.0271222, PMID: 35802731 PMC9269923

[B21] Farsi N. J. Baharoon A. H. Jiffri A. E. Marzouki H. Z. Merdad M. A. Merdad L. A. (2021). Human papillomavirus knowledge and vaccine acceptability among male medical students in Saudi Arabia. Hum. Vaccines Immunother. 17, 1968–1974. doi: 10.1080/21645515.2020.1856597, PMID: 33522406 PMC8189128

[B22] Gamaoun R. (2018). Knowledge, awareness and acceptability of anti-HPV vaccine in the Arab states of the Middle East and North Africa Region: a systematic review. East. Mediterr. Health J. 24, 538–548. doi: 10.26719/2018.24.6.538, PMID: 30079949

[B23] Ghojazadeh M. Azar Z. F. Saleh P. Naghavi-Behzad M. Azar N. G. (2012). Knowledge and attitude of Iranian University students toward human papilloma virus. Asian Pac. J. Cancer Prev. APJCP. 13, 6115–6119. doi: 10.7314/APJCP.2012.13.12.6115, PMID: 23464415

[B24] Haddad S. F. Kerbage A. Eid R. Kourie H. R. (2022). Awareness about the human papillomavirus (HPV) and HPV vaccine among medical students in Lebanon. J. Med. Virol. 94, 2796–2801. doi: 10.1002/jmv.27509, PMID: 34877678

[B25] Hamdi S. (2017). The impact of teachings on sexuality in Islam on HPV vaccine acceptability in the Middle East and North Africa region. J. Epidemiol. Glob. Health 7, S17–S22., PMID: 29801588 10.1016/j.jegh.2018.02.003 PMC7386444

[B26] HPV Information Centre Complementary Data on Cervical Cancer Prevention. Available online at: https://hpvcentre.net/.

[B27] Khalil J. Boutros S. Hassoun A. Hallit S. Barakat H. (2023). Human papillomavirus vaccine knowledge and conspiracy beliefs among secondary school students in Lebanon. BMC Pediatr. 23, 363. doi: 10.1186/s12887-023-04177-w, PMID: 37454098 PMC10349416

[B28] Knobel P. Zhao X. White K. M. (2022). Do conspiracy theory and mistrust undermine people’s intention to receive the COVID-19 vaccine in Austria? J. Community Psychol. 50, 1269–1281. doi: 10.1002/jcop.22714, PMID: 34551127 PMC8656288

[B29] Kombe Kombe A. J. Li B. Zahid A. Mengist H. M. Bounda G. A. Zhou Y.. (2021). Epidemiology and burden of human papillomavirus and related diseases, molecular pathogenesis, and vaccine evaluation. Front. Public Health 8. doi: 10.3389/fpubh.2020.552028/full, PMID: 33553082 PMC7855977

[B30] Mahafzah A. M. Al-Ramahi M. Q. Asa’d A. M. El-Khateeb M. S. (2008). Prevalence of sexually transmitted infections among sexually active Jordanian females. Sex. Transm. Dis. 35, 607–610. doi: 10.1097/OLQ.0b013e3181676bbd, PMID: 18434942

[B31] Mehta S. Rajaram S. Goel G. Goel N. (2013). Awareness about Human Papilloma Virus and its Vaccine Among Medical Students. Indian J. Community Med. 38, 92–94. doi: 10.4103/0970-0218.112438, PMID: 23878421 PMC3714948

[B32] Murewanhema G. Moyo E. Dzobo M. Mandishora-Dube R. S. Dzinamarira T. (2024). Human papilloma virus vaccination in the resource-limited settings of sub-Saharan Africa: Challenges and recommendations. Vaccine X. 20, 100549. doi: 10.1016/j.jvacx.2024.100549, PMID: 39263366 PMC11388769

[B33] Nesser W. Ayodele O. (2023). Human papilloma virus knowledge among university students, staff, and faculty in the state of Indiana during 2016, 2019, and 2022. J. Community Health 48, 718–723. doi: 10.1007/s10900-023-01210-y, PMID: 36988774 PMC10052299

[B34] Oh K. M. Alqahtani N. Chang S. Cox C. (2023). Knowledge, beliefs, and practice regarding human papillomavirus (HPV) vaccination among American college students: Application of the health belief model. J. Am. Coll. Health J. ACH. 71, 2329–2338. doi: 10.1080/07448481.2021.1967362, PMID: 34586013

[B35] Orji C. Brown C. M. Barner J. Moczygemba L. Morales-Campos D. (2025). Determinants of Human Papillomavirus (HPV) vaccination intentions among young adult college students using the theory of planned behavior. J. Am. Coll. Health J. ACH. 73, 2660–2673. doi: 10.1080/07448481.2024.2325935, PMID: 38466334

[B36] Othman A. Abuidhail J. Shaheen A. Langer A. Gausman J. (2022). Parents’ perspectives towards sexual and reproductive health and rights education among adolescents in Jordan: content, timing and preferred sources of information. Sex. Educ. 22, 628–639. doi: 10.1080/14681811.2021.1975671

[B37] Pertwee E. Simas C. Larson H. J. (2022). An epidemic of uncertainty: rumors, conspiracy theories and vaccine hesitancy. Nat. Med. 28, 456–459. doi: 10.1038/s41591-022-01728-z, PMID: 35273403

[B38] Porras C. Tsang S. H. Herrero R. Guillén D. Darragh T. M. Stoler M. H.. (2020). Efficacy of the bivalent HPV vaccine against HPV 16/18-associated precancer: long-term follow-up results from the Costa Rica Vaccine Trial. Lancet Oncol. 21, 1643–1652. doi: 10.1016/S1470-2045(20)30524-6, PMID: 33271093 PMC8724969

[B39] Qaqish A. Abdo N. Abbas M. M. Saadeh N. Alkhateeb M. Msameh R.. (2023). Awareness and knowledge of physicians and residents on the non-sexual routes of human papilloma virus (HPV) infection and their perspectives on anti-HPV vaccination in Jordan. PloS One 18, e0291643. doi: 10.1371/journal.pone.0291643, PMID: 37819974 PMC10566688

[B40] Rani U. Darabaner E. Seserman M. Bednarczyk R. A. Shaw J. (2022). Public education interventions and uptake of human papillomavirus vaccine: A systematic review. J. Public Health Manag. Pract. JPHMP. 28, E307–E315. doi: 10.1097/PHH.0000000000001253, PMID: 33208719

[B41] Rashid S. Labani S. Das B. C. (2016). Knowledge, awareness and attitude on HPV, HPV vaccine and cervical cancer among the college students in India. PloS One 11, e0166713. doi: 10.1371/journal.pone.0166713, PMID: 27861611 PMC5115771

[B42] Reno J. E. Thomas J. Pyrzanowski J. Lockhart S. O’Leary S. T. Campagna E. J.. (2019). Examining strategies for improving healthcare providers’ communication about adolescent HPV vaccination: evaluation of secondary outcomes in a randomized controlled trial. Hum. Vaccines Immunother. 15, 1592–1598. doi: 10.1080/21645515.2018.1547607, PMID: 30433845 PMC6746480

[B43] Sallam M. Dababseh D. Yaseen A. Al-Haidar A. Ettarras H. Jaafreh D.. (2022). Lack of knowledge regarding HPV and its relation to oropharyngeal cancer among medical students. Cancer Rep. 5, e1517. doi: 10.1002/cnr2.1517, PMID: 34291614 PMC9327668

[B44] Sarhan M. M. Kelly J. El-Farra N. Rashid M. A. (2025). Collaborating for change: reimagining medical education in Jordan through international partnerships. Discov. Educ. 4, 2. doi: 10.1007/s44217-024-00395-1

[B45] Shah S. F. A. Ginossar T. Bentley J. M. Zimet G. McGrail J. P. (2021). Using the Theory of Planned behavior to identify correlates of HPV vaccination uptake among college students attending a rural university in Alabama. Vaccine 39, 7421–7428. doi: 10.1016/j.vaccine.2021.10.082, PMID: 34772544

[B46] Solis-Torres N. Braverman-Diaz I. Rivera-Morales L. A. Perez-Sanchez J. J. Perez-Bravo V. S. Neris-Sanchez A. J.. (2024). Medical students’ knowledge about human papillomavirus (HPV), HPV vaccine and head and neck cancer. Hum. Vaccines Immunother. 20, 2344248. doi: 10.1080/21645515.2024.2344248, PMID: 38659106 PMC11057669

[B47] Sung H. Ferlay J. Siegel R. L. Laversanne M. Soerjomataram I. Jemal A.. (2021). Global cancer statistics 2020: GLOBOCAN estimates of incidence and mortality worldwide for 36 cancers in 185 countries. CA Cancer J. Clin. 71, 209–249. doi: 10.3322/caac.21660, PMID: 33538338

[B48] Teeluck H. K. Bholoa D. K. Keenoo B. S. (2022). Level of awareness, attitude and perception about human papilloma virus vaccine among University of Mauritius students. Afr. J. Reprod. Health 26, 41–48., PMID: 37585084 10.29063/ajrh2022/v26i12.5

[B49] Wang W. Lyu J. Li M. Zhang Y. Xu Z. Chen Y.. (2021). Quality evaluation of HPV vaccine-related online messages in China: a cross-sectional study. Hum. Vaccines Immunother. 17, 1089–1096. doi: 10.1080/21645515.2020.1814095, PMID: 33054581 PMC8018388

[B50] Wang R. Pan W. Jin L. Huang W. Li Y. Wu D.. (2020). Human papillomavirus vaccine against cervical cancer: Opportunity and challenge. Cancer Lett. 471, 88–102. doi: 10.1016/j.canlet.2019.11.039, PMID: 31812696

[B51] World Health Organization (2024). Human papillomavirus and cancer. Available online at: https://www.who.int/news-room/fact-sheets/detail/human-papilloma-virus-and-cancer (Accessed June 5, 2025).

[B52] Yacouti A. Elkhoudri N. El Got A. Benider A. Hadrya F. Baddou R.. (2022). Awareness, attitudes and acceptability of the HPV vaccine among female university students in Morocco. PloS One 17, e0266081. doi: 10.1371/journal.pone.0266081, PMID: 35395019 PMC8993020

