# Supplementary material for: Knowledge and attitudes of medical students regarding human papilloma virus infection and vaccine: cross-sectional study from Jordan
Source: Front Cell Infect Microbiol. 2025 Sep 18;15:1657090. doi: 10.3389/fcimb.2025.1657090 (PMC12488729; doi:10.3389/fcimb.2025.1657090)
Supplement: Supplementary file 1 [file Supplementaryfile1.docx]

| 'You are invited you to fill out a form: |
| --- |
|  |
| [Awareness and knowledge of medical students about Human Papilloma Virus (HPV) Infection in Jordan.](https://docs.google.com/forms/d/e/1FAIpQLSfZ5kKy8-TA-3FWAAkER1AX_vJs3Jz7Nj7aLgcJ84QGvE_kEQ/viewform?vc=0&c=0&w=1&flr=0&usp=mail_form_link) |
|  |
| This scientific research aims to assess the knowledge of medical students about Human Papilloma Virus (HPV) Infection and prevention strategies in Jordan.  Your participation is voluntary, anonymous and confidential.  The data will be analyzed by the research team to provide data for decision making to prevent and control HPV in Jordan. If you are a medical student and you voluntary agree to participate in this research, please answer the below questions |
|  |
| Top of Form  **In which year are you in medical school? ***   - 1st year - 2nd year - 3rd year - 4th year - 5th year - 6th year   **In which university is your medical school? ***   - The University of Jordan (JU) - Jordan University of Science & Technology (JUST) - The Hashemite University (HU) - Yarmouk University (YU) - Mu'tah University (MU) - Al- Balqa' Applied University (BAU)   **What is your gender? ***   - Male - Female   **What is your martial status? ***   - Married - Unmarried   **What is your age? ***   - <20 - 20-39 - >39   **What is your nationality? ***   - Jordanian - Non-Jordanian   **If you choose "non-Jordanian", please write down what is your nationality?**  **HPV Knowledge**  **Have you heard about HPV before? ***   - Yes - No - I don't know   **Do you think HPV is common in Jordan? ***   - Yes - No - I don't know   **Which people does HPV infect? ***   - Males - Females - Both males and females - I don't know   **Can HPV infection be asymptomatic? ***   - Yes - No - I don't know   **Do you think HPV infection is transmitted through sexual intercourse only? ***   - Yes - No - I don't know   **If you didn't answer "yes", which of the following routes is an HPV transmission route?**   \|  \| Yes \| No \| I don't know \| \| --- \| --- \| --- \| --- \| \| Skin-skin contact \|  \|  \|  \| \| Skin-mucosa contact \|  \|  \|  \| \| Mother to fetus \|  \|  \|  \| \| Contact with contaminated medical equipment \|  \|  \|  \| \| Contact with contaminated water \|  \|  \|  \| \| Self-inoculation \|  \|  \|  \|   **The major risk of having HPV infection in females is ______ ***   - Cancer - Genital warts - Skin warts - I don't know   **The major risk of having HPV infection in males is ______ ***   - Cancer - Genital warts - Skin warts - I don't know   **Can HPV infection be treated? ***   - Yes - No - I don't know   **How often do you think cervical cytology should be performed? ***   - Once every 3 years - Once every year - Once every 6 months - I don't know   **HPV Vaccine**  **Have you heard about vaccine against HPV? ***   - Yes - No - I don't know   **Can the vaccine be protective if the person is already infected with HPV? ***   - Yes - No - I don't know   **Do you think that someone who is already suffering from genital warts associated with HPV infection has indications for being vaccinated against HPV? ***   - Yes - No - I don't know   **Are there any indications for vaccinating boys?         ***   - Yes - No - I don't know   **When is the best time to get vaccinated against HPV? ***   - Children less than 8 years of age - Children aged 9-13 or before marriage/sexual activity - After marriage/sexual activity - I don't know   **How many doses of vaccine against HPV do you think are given in a life time? ***   - One does - Three doses - Five doses   **Can adults aged 14- 45 get vaccination against HPV to protect against genital warts and/or different types of HPV that can cause cancer. ***   - Yes - No - I don't know   **Is the vaccine available in Jordan? ***   - Yes - No - I don't know   **If the vaccine is available in Jordan, would you take the vaccine? ***   - Yes - No - I don't know   **If no, the reason is which of the below?**   \|  \| Yes \| No \| I don't know \| \| --- \| --- \| --- \| --- \| \| you are virgin \|  \|  \|  \| \| you have no extra-marital sex activity \|  \|  \|  \| \| you are not a female \|  \|  \|  \| \| you doubt the protective efficacy of the vaccine \|  \|  \|  \| \| you are worried the vaccine is not safe \|  \|  \|  \| \| you cannot afford the cost of the vaccine \|  \|  \|  \|   **If you have another reason for the previous question other than the listed, please write it down.**  **If the vaccine is available in Jordan for free, would you recommend taking the vaccine to a family member? ***   - Yes - No - I don't know   **If no, the reason is which of the below?**   \|  \| Yes \| No \| I don't know \| \| --- \| --- \| --- \| --- \| \| you doubt the protective efficacy of the vaccine \|  \|  \|  \| \| you are worried the vaccine is not safe \|  \|  \|  \| \| For cultural and religious reasons \|  \|  \|  \|   **If you have another reason for the previous question other than the listed, please write it down.**  **If the vaccine is available in Jordan for free, would you recommend the vaccine for your patients? ***   - Yes - No - I don't know   **If no, the reason is which of the below?**   \|  \| Yes \| No \| I don't know \| \| --- \| --- \| --- \| --- \| \| you doubt the protective efficacy of the vaccine \|  \|  \|  \| \| you are worried the vaccine is not safe \|  \|  \|  \| \| For cultural and religious reasons \|  \|  \|  \|   **If you have another reason for the previous question other than the listed, please write it down.**  **What is the estimated percentage of cervical cancers that could be prevented by HPV (human papillomavirus) vaccination? ***   - 25% - 50% - 60% - 90% - I don't know   **Do you agree that vaccination against HPV should be obligatory in Jordan? ***   - Yes - No - I don't know   **What were your sources of knowledge about the vaccine against HPV? ***   - Friends - Family - Medical Education - Social Media - Self-reading   Bottom of Form |
